# scMRMA: single cell multiresolution marker-based annotation

**DOI:** 10.1093/nar/gkab931

**Published:** 2021-10-14

**Authors:** Jia Li, Quanhu Sheng, Yu Shyr, Qi Liu

**Affiliations:** Department of Biostatistics, Vanderbilt University Medical Center, Nashville, TN 37203, USA; Center for Quantitative Sciences, Vanderbilt University Medical Center, Nashville, TN 37232, USA; Department of Biostatistics, Vanderbilt University Medical Center, Nashville, TN 37203, USA; Center for Quantitative Sciences, Vanderbilt University Medical Center, Nashville, TN 37232, USA; Department of Biostatistics, Vanderbilt University Medical Center, Nashville, TN 37203, USA; Center for Quantitative Sciences, Vanderbilt University Medical Center, Nashville, TN 37232, USA; Department of Biostatistics, Vanderbilt University Medical Center, Nashville, TN 37203, USA; Center for Quantitative Sciences, Vanderbilt University Medical Center, Nashville, TN 37232, USA

## Abstract

Single-cell RNA sequencing has become a powerful tool for identifying and characterizing cellular heterogeneity. One essential step to understanding cellular heterogeneity is determining cell identities. The widely used strategy predicts identities by projecting cells or cell clusters unidirectionally against a reference to find the best match. Here, we develop a bidirectional method, scMRMA, where a hierarchical reference guides iterative clustering and deep annotation with enhanced resolutions. Taking full advantage of the reference, scMRMA greatly improves the annotation accuracy. scMRMA achieved better performance than existing methods in four benchmark datasets and successfully revealed the expansion of CD8 T cell populations in squamous cell carcinoma after anti-PD-1 treatment.

## INTRODUCTION

Single-cell RNA sequencing provides an unprecedented opportunity to characterize cellular heterogeneity in a variety of biological contexts by profiling transcriptomes of thousands to millions of cells simultaneously (1–3). One essential but challenging step to understanding cellular heterogeneity is determining cell identities (4). Initially, manual annotation was employed, but it is labor intensive, unreproducible and unscalable for large-scale datasets (5). The recent explosive growth in single-cell RNA sequencing has prompted the development of automatic cell-type annotation methods (6). Automatic cell type annotation methods use a common strategy which projects a query against a reference unidirectionally to find the best match. The query is either a single cell or a cell cluster, and a reference is a collection of known cell types characterized by marker genes or transcriptomic profiles (7).

Cell annotation methods either map single cells against a reference, such as SingleR (8), scMATCH (9) and Garnett (10), or annotate pre-computed cell clusters based on average expression profiles such as scCATCH (11), SCSA (12) and Alona (13). Due to high technical and biological noise inherent in single-cell RNA sequencing data, sometimes transcriptionally similar cells are annotated to different cell types by single-cell query methods. Cluster-based query is robust against noise; however, it is difficult to choose an optimal resolution for cell clustering. Low-resolution clustering impairs discriminative ability on specific cell types, while high-resolution may be unnecessary and sometimes introduce noise. Moreover, single-resolution clustering cannot capture global and local biological variances simultaneously.

Some methods use marker genes to define known cell types in their reference, such as Garnett (10), CellAssign (14) and scCATCH (11). Those markers are panels of genes that uniquely identify cell types through literature or expert-curated knowledge databases like CellMarker (15), PanglaoDB (16) and CancerSEA (17). Other methods employ expression profiles from pre-annotated and purified cell types quantitatively, such as SingleR (8), CHETAH (18) and scMAP (19). The popular transcriptome-based references are HCA (20), MCA (21) and FANTOM5 (22). Most methods assemble their references by a flat structure, where each cell type is treated equally and independently (8,11,14). The flat structure works well for distinguishing broad cell types, while lacks the resolution to distinguish more specific subpopulations due to inability to reflect rich relationships between cell types. CHEATH (18), Garnett (10) and CellO (23), in contrast, organize cell types hierarchically from broad to very specific, improving discrimination for specific cell types.

We developed the ‘single cell Multiresolution Marker-based Annotation’ (scMRMA) algorithm, a bidirectional method that not only maps cell clusters against a hierarchical reference but the reference also guides cell clustering to identify distinctive subgroups for annotation. Under the guidance of a hierarchical reference, the resolution for cell clustering gets higher for deeper annotation. This multiresolution strategy enables the detection of subtle difference and improves discriminating ability for very specific cell types. Using four benchmark datasets from various tissues and platforms, we demonstrated that scMRMA outperformed other methods in annotation accuracy. With high discriminative ability, scMRMA successfully uncovered the expansion of CD8 T cells after anti-PD-1 treatment in squamous cell carcinoma.

## MATERIALS AND METHODS

### scRNAseq data preprocessing

scMRMA takes a gene-by-cell raw count matrix as input, normalizes by the total number of reads, scales values to 10 000 and log-transforms after the addition of 1. The top 2000 most variable genes are identified by modeling mean-variance relationship using local polynomial regression (loess, span = 0.3) (24). The normalized and log-transformed gene expression of the top 2000 most variable genes are then scaled and used for principal component analysis. The top 50 principal components are used for clustering.

### Iterative cluster-and-annotate of scMRMA

scMRMA employs an iterative cluster-and-annotate strategy to capture both global and local variances across broad and specific cell types, where a hierarchical reference guides the label assignment. First, query cells are clustered and annotated to reference cell types at the first level. Cells assigned to the same cell type are further clustered if there are more than one child nodes under this cell type. In this round of clustering, highly variable genes are rechosen, principal component analyses are reperformed, and the cells belonging to the same parental cell type are re-clustered. Through this process, the focus shifts from global variance among all cells to local variances within the cells belonging to the same parental cell type, which is expected to obtain an accurate sub-clustering. This re-clustering procedure will be repeated until reaches to a leaf node or fails to assign cells to any intermediate nodes.

A graph-based approach is used for clustering, where cells are embedded in a graph structure, and then partitioned into highly connected ‘communities’. Briefly, a *K*-nearest neighbor (KNN, default *k* = 20) graph for each cell is built on the Euclidean distance calculated by the top 50 PCs (25). A shared nearest neighbor (SNN) graph is constructed by calculating the neighborhood overlap between every cell and its *K*-nearest neighbors (26,27). The Louvain community detection method is used to identify highly connected ‘communities’ (cell clusters) by optimizing the modularity function on the SNN graph (28).

### Cell type annotation

scMRMA maps cell clusters to a hierarchical reference to determine their identities. The cell clusters are represented by their average expression profiles, while the reference is characterized by a collection of marker genes. For a given cluster, scMRMA uses the approach described in (16) to calculate an activity score for each cell type by summing the weighted expression of its marker genes.

(1) $$\begin{equation*}{\rm{A}}{{\rm{S}}_{{\rm{c}},{\rm{t}}}}{\rm{}} = {\rm{}}\frac{{\mathop \sum \nolimits_{{{i}} = {\rm{}}1}^{\rm{N}} \left( {{\rm{Ex}}{{\rm{p}}_{{t_i},{\rm{c}}}}{\rm{*}}{{\rm{w}}_{{t_i}}}} \right)}}{{{{\rm{N}}^{\rm{r}}}}},\;t = \left( {{t_1},{t_2}, \ldots {t_N}} \right)\end{equation*}$$

(2) $$\begin{equation*}{{\rm{w}}_{{t_i}}} = {\rm{}}1 + {\rm{}}\sqrt {\frac{{\max \left( {{f}} \right) - {{{f}}_{{t_i}}}}}{{\max \left( {{f}} \right) - {\rm{min}}\left( {{f}} \right)}}} \end{equation*}$$

${\rm{A}}{{\rm{S}}_{{\rm{c}},{\rm{t}}}}$ is the activity score of the cell type *t* for a given cell cluster *c*, where the cell type *t* is a collection of *N* marker genes (${t_1},{t_2}, \ldots {t_N}).\;{\rm{Ex}}{{\rm{p}}_{{t_i},{\rm{c}}}}$ is the average expression of gene ${t_i}$ in the cluster *c*, and ${{\rm{w}}_{{t_i}}}$ is the weights of gene ${t_i}$. The gene is weighted by its frequency ${{{f}}_{{t_i}}}\;$in the reference, where common markers is down weighted and specific ones is up weighted (Equation 2). *r* is a constant factor to adjust the score for the total number of markers in the cell type, which is set to 0.3 in the original literature (16). Since scMRMA organizes the reference from very broad (the first level) to specific (the leaf node), broad cell types include all the marker genes from their child nodes. Therefore, the number of marker genes vary a lot at the broad level. To adjust the effect caused by the number of marker genes, scMRMA uses different parameters to calculate activity scores at the first level and other levels. The average expression profile and *r* of 0.6 are used at the first level, while the *z*-transformed expression and *r* of 0.3 are utilized at other levels. Since the first level consists of a large number of genes and distinct cell types, many genes are not expressed simultaneously. Those unexpressed genes would pulled down activity scores to a small value, even to negative, if *z*-transformed expression were used. Therefore, the average gene expression without z-transformation was used at the first level. In contrast, the lower level includes specific cell types and fewer number of genes. In this case, *z*-scoring is beneficial to generate discriminative activity scores. Besides cell activity score, a *P* value is computed for each cell type by using one-sided Fisher’s exact test. The cell cluster is assigned to the cell type with the highest activity score if the *P* value is <0.05 for the ‘winner’. Otherwise, the cluster is set as ‘unassigned’.

### The hierarchical reference

To build a hierarchical reference, we started from PanglaoDB, a maker-based database consisting of 178 cell types involving 3764 (human) and 3825 (mouse) marker genes in a flat structure (16). We used Cell Ontology (29,30) to reorganize the 176 cell types into a hierarchical structure. Cell ontology incorporates phenotypic information of cell types and is more comprehensive in its cellular detail than other resources. It supports the consistent representation of cell types across different levels of anatomical granularity, such as tissues and organs (29,30). The hierarchical PanglaoDB has four levels (Figure 2A). There are seven broad cell types at the first level, Connective tissue cell (CL_0002320), Epithelial cell (CL_0000066), Hematopoietic cell (CL_0000988), Muscle cell (CL_0000187), Neural cell (CL_0002319), Precursor cell (CL_0011115) and Somatic cell (CL_0002371). There are 51, 109 and 59 cell types at the second, third and fourth levels, respectively (Figure 2A). scMRMA uses the hierarchical PanglaoDB as the default reference. Besides, scMRMA provides another reference named TcellAI, which contains 22 subtypes of T cells in a two-level hierarchical structure (Figure 7A). TcellAI is a part of ImmuCellAI, which was originally collected for cancer immune cells (31). Therefore, TcellAI is very useful for annotating specific T-cell populations in cancer. In addition, scMRMA accepts user-defined references as well.

### Datasets

All the datasets and cell type annotation were downloaded from GEO. Four datasets, GSM2486333 (32), GSE130148 (33), GSE84133 (34) and GSM3580745 (35), were used for evaluating performance on annotation accuracy. They are single cells collected from human peripheral blood monoclonal cells, human lung tissue, human pancreatic islets and mouse cerebral cortex and generated by Seq-Well, drop-seq, inDrops and 10× technologies, respectively. One dataset, GSE123813 (36), was used to demonstrate the ability of scMRMA to reveal the change of cell compositions. It was derived from single-cell squamous cell carcinoma (SCC) before and after anti-PD1 therapy treatment dataset by 10× technology.

Cells with >20% mitochondria reads, or <500 UMI reads, or <200 or >6000 genes expressed were excluded. Filtering low quality cells before annotations is necessary to avoid misleading biological conclusions and inaccurate annotations. Cells without annotation in the literatures were removed. After filtering, there were 3383 cells and five immune cell types left in the human Peripheral Blood Mononuclear Cell (PBMC) dataset (32), 5902 cells and 12 cell types in the human lung dataset (33), 8556 cells and 13 cell types in the human pancreas dataset (34), 3916 cells and 12 cell types in the mouse brain dataset (35) and 26 015 cells and 10 cell types in the human SCC dataset (36).

### Comparison methods

scMRMA was compared with four annotation algorithms: CellAssign (14), Garnett (10), scCATCH (11) and SingleR (8). CellAssign, Garnett and SingleR annotate single cells, while scCATCH predicts identities of cell clusters. Garnett, scCATCH and CellAssign use markers-based reference, while SingleR depends on transcriptome-based reference. Among the four methods, only Garnett organizes the reference in a hierarchical rather than a flat structure.

Garnett (10) provides supervised classification, which first uses a marker-based reference to select representative cells in a dataset and then trains a classifier to predict cell types for other cells based on their similarity with representative cells. Garnett has two options, using a pre-trained classifier, or generating your own classifier. Since pre-trained classifiers exist for human PBMC (hsPBMC), human lung (hsLung) (10,37) and mouse brain (mmBrain) (10,38), they were used for cell type annotation on the corresponding datasets. For the human pancreas dataset without a pre-trained classifier, we generated our own classifier using the marker genes in the literature (34) and default parameters. Seurat clusters with a resolution 0.8 were used to extend classifications to additional cells in the same cluster.

CellAssign (14) offers a probabilistic model that leverages a marker-based reference for cell type assignment. Since CellAssign does not provide a pre-defined list of marker genes, we used marker genes in the pre-trained/trained Garnett classifiers (10) as the marker file input for CellAssign. The marker file was transformed to a gene-by-cell-type binary matrix, whose entries were set to 1 if a gene is a specific marker for a given cell type and 0 otherwise. Default parameters were used to run CellAssign.

scCATCH (11) annotates cell clusters through a tissue-specific cell taxonomy reference databases CellMatch (11,15,17,21) and evidence-based score. Seurat clusters with a resolution 0.8 were used as the cluster input. The parameter match_CellMatch was set as ‘TRUE’ and the parameters ‘species’ and ‘tissue’ were set to the corresponding species and tissues. Other parameters were kept as default.

SingleR (8) correlates single-cell expression profile with reference transcriptome of pure cell types profiled by microarray and bulk RNA-seq to assign cell identity. There are multiple reference transcriptomes available, including Human Primary Cell Atlas dataset (HumanPrimaryCellAtlasData) (39), Encode and Blueprint Epigenomics dataset (BlueprintEncodeData) (40,41) and Mouse RNA-seq dataset (MouseRNAseqData) (42). The references achieving the highest annotation accuracy were used to report the result. Specifically, HPCA was used for the annotation of the human PBMC and lung datasets, while Encode was utilized for the annotation of the human pancreas and SCC datasets. MouseRNAseqData (42) was used for the classification of the mouse brain dataset.

### Annotation accuracy

Annotation accuracy was defined as the number of cells assigned with the same cell type as in the literature. Some cell types were combined into a broad cell type if there were only very limited number of cells or removed due to lack of specific cell types defined in all references. For example, GABAergic neurons and glutamatergic neurons were combined as neurons, while multiplets (*n* = 69) labeled by the original study were removed in the mouse brain dataset (35). Mast cells, macrophages and T cells were combined as hematopoietic cells, quiescent stellate cells and activated stellate cells were combined as Stellate cells, while Schwann cells (*n* = 13) were removed in the human pancreas dataset. CD4 and CD8 annotated in the human PBMC dataset were combined into T cells. Secretory and transformed epithelium were combined as epithelium, while Lymphatic cells (*n* = 105) were removed in the human lung dataset.

## RESULTS

### Overview of scMRMA algorithm

**Figure 1.**
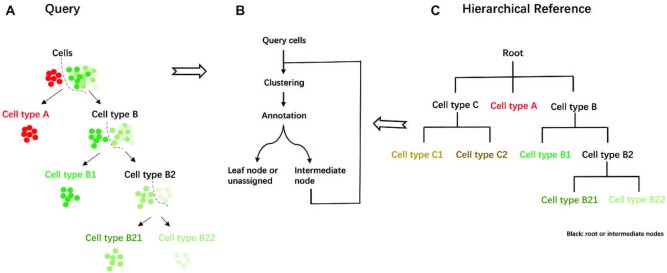
Overview of scMRMA algorithm. (**A**) The query cells were clustered and then annotated from broad cell types to specific ones. The color denotes cell types, where cells with the same color belong to the same cell type. (**B**) The iterative cluster-and-annotate procedure of scMRMA and (**C**) the Hierarchical reference. Black represents root or intermediate nodes, while colorful nodes are leaf nodes.

scMRMA maps cell clusters against a collection of known cell types characterized by marker genes and organized in a hierarchical structure for cell type annotation (Figure 1). scMRMA not only projects cell clusters against the reference but also uses the hierarchical reference to guide the subcluster refinement when necessary. Rather than fixed and pre-computed cell clusters, scMRMA refines cell clusters every time the annotation moves to a deeper level. Specifically, when there are more than one child nodes under a parental cell type, it suggests the necessity of a closer look into those cells assigned to the parental type for a deep annotation. To achieve this, cells belonging to the parental node are re-clustered to capture the difference within them. Then, the refined subclusters are mapped against the child nodes to find the best match using only marker genes that discriminate them. scMRMA repeats this process that traverses the hierarchical tree until it reaches a leaf node or halts at an intermediate node due to assignment failure (‘unassigned’) (Figure 1B). For example, cells are annotated to cell type A and B at the first step (Figure 1A). Cell type A is a leaf node; therefore, cells predicted as type A come to their final annotation without an enhanced resolution clustering. In contrast, cell type B includes two subtypes B1 and B2 (Figure 1C); thus, cells annotated to cell type B go through a re-clustering procedure to capture the variance within them. The refined two clusters are annotated as cell type B1 and B2, respectively. Repeatedly, only cells annotated as B2 are re-clustered and then assigned to B21 and B22 types (Figure 1A). Unlike most approaches using a single and uniform resolution for cell clustering, scMRMA employs an iterative cluster-and-annotation strategy which cells are grouped by multiple resolutions under the guidance of a hierarchical reference. Cells assigned to deeper annotated cell types undergo multiple clustering steps, which results in higher resolutions for capturing the minor difference. The multiresolution framework of scMRMA allows the focus shifting from genes distinguishing broad cell types to those differentiating sibling cell types.

**Figure 2.**
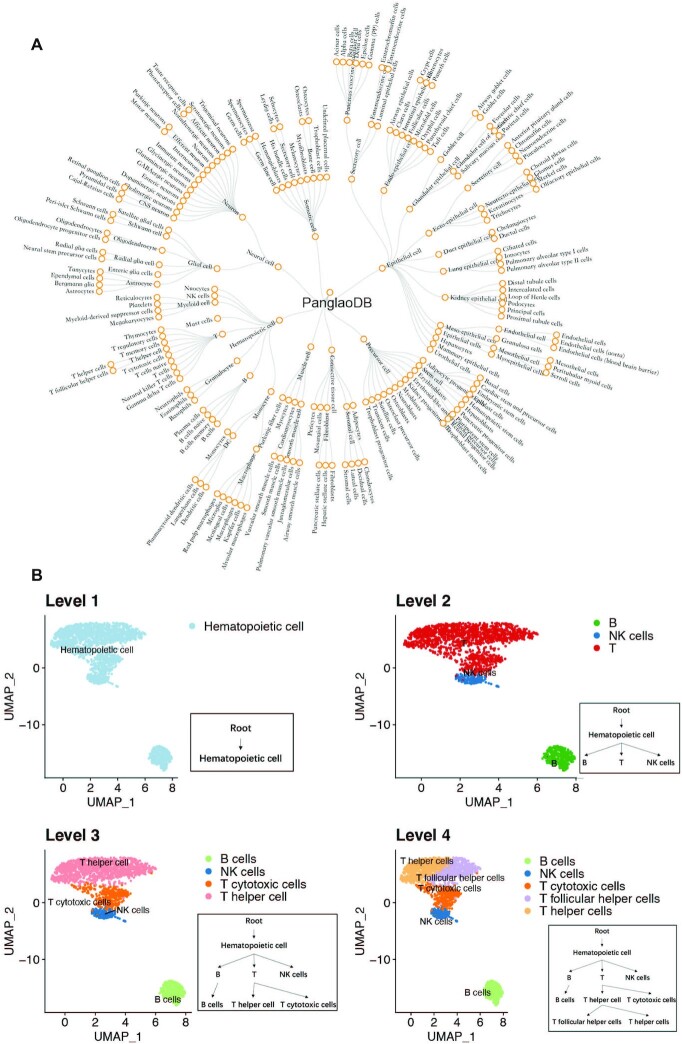
A toy example of scMRMA annotation process. (**A**) The hierarchical PanglaoDB reference in a four-level structure. (**B**) A toy example showing annotation from the first level to the fourth level based on the hierarchical PanglaoDB reference. The color represents cell types.

We demonstrated the iterative cluster-and-annotate procedure of scMRMA by a toy example. In the example, the hierarchical PanglaoDB was used as the reference, whose structure was illustrated in Figure 2A. First, all cells were clustered and annotated to ‘hematopoietic cell’ based on marker genes at the first level. Second, all cells were re-clustered and annotated to ‘B’, ‘T’ and ‘NK cells’ using marker genes differentiating cell types at the second level. Third, cells predicted as ‘NK cells’ reach their final annotation since ‘NK cells’ is a leaf node. Cells classified as ‘B’ and ‘T’ were re-clustered separately. The refined clusters were annotated to the third level according to marker genes discriminating sibling nodes in ‘B’ and ‘T’, respectively. As a result, cells belonging to ‘B’ were annotated as ‘B cells’ (leaf node). Cells belonging to ‘T’ were further classified into ‘T helper cell’ and ‘T cytotoxic cells’. Finally, cells predicted as ‘T helper cell’ underwent a further re-clustering. Some re-clustered cells were annotated as ‘T follicular helper cells’, while others were predicted to be ‘T helper cells’ (Figure 2B).

### scMRMA outperforms other methods

**Figure 3.**
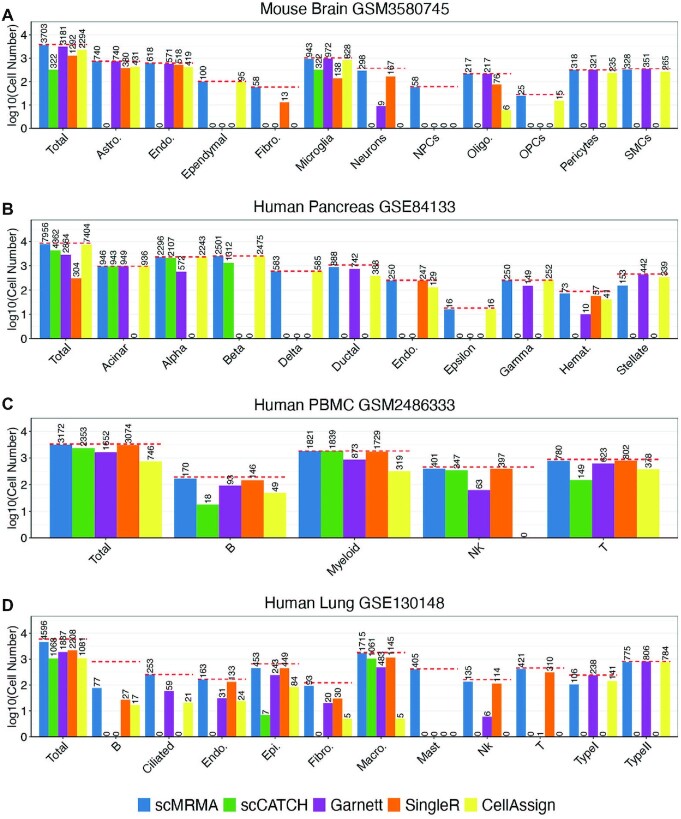
Comparative analysis of annotation accuracy. (**A**) The number of cells assigned correctly by scMRMA, scCATCH, Garnett, SingleR and CellAssign in the mouse brain dataset; *Y-*axis shows the number of cells in the log10 scale, while *X*-axis is the total and each individual cell type. The ‘Gold-standard’ annotation from the literature was represented by red dashed lines. (B–D) The same measurement as in (**A**) from the human pancreas (**B**), PBMC (**C**) and lung datasets (**D**).

We benchmarked scMRMA against Garnett (10), CellAssign (14), scCATCH (11) and SingleR (8) in terms of annotation accuracy on four scRNA-seq datasets. These datasets were collected from brain, pancreas, lung and blood from human or mouse and generated by various platforms (32–35) (details in Materials and Methods section). scMRMA achieved the best performance in all four datasets, with annotation accuracy of 94.6%, 93%, 93.8% and 77.9% for the mouse brain, and the human pancreas, PBMC and lung datasets, respectively (Figure 3). Moreover, scMRMA predicted most accurately in almost every individual cell type, including dominant and rare cell types (Figure 3 and Supplementary Figures S1–S4).

Compared to scMRMA achieving the most accurate annotation in all four datasets, the performance of Garnett, CellAssign, scCATCH and SingleR is dataset-dependent. For the mouse brain dataset, scMRMA predicted 3703 out of 3916 cells correctly (94.6%), followed by Garnett which provided the right annotation for 3181 cells (87.3%). Garnett misclassified ependymal cells, fibroblasts and Oligodendrocyte Progenitor Cells (OPCs) as ‘unknown’ although those cell types were included in its reference. Besides, it only detected nine neurons and missed most of them (assigned as ‘unknown’). Using the same reference as Garnett, CellAssign only identified 2294 cells successfully (59.5%). It assigned most ependymal cells correctly, but failed in fibroblasts, neurons and oligodendrocytes. scCATCH only predicted 322 microglia cells correctly and failed to annotate all other cells although its reference includes all those cell types (Supplementary Figure S1). Out of 8556 cells in the human pancreas dataset, scMRMA labeled 7956 cells correctly (93%), followed by CellAssign for 7404 cells (86.5%) and scCATCH for 4362 cells (51%) (Figure 3). scCACTH misclassified delta, ductal, endothelial, hematology and stellate cells although they were included in its reference. Its failures in Epsilon and Gamma cells, however, were due to a lack of those cell types in its reference. Garnett failed to recognize Beta, Delta, Endothelial, Epsilon cells, and most of Alpha cells although it used the same reference as CellAssign. SingleR performed well in endothelial and hematology cells but failed in other cell types due to a lack of reference profiles of those cell types (Supplementary Figure S2). Hematology cells included three distinct cell types: mast cells, macrophages and T cells. scMRMA successfully assigned mast cells and macrophages but failed in annotating T cells (Supplementary Figure S3). The reason for its failure was due to incorrect clustering of rare T cells (only seven cells). With the default *k* value to build the *k*-nearest neighbor graph (*k* = 20), T cells were clustered with mast cells, leading to false annotation. scMRMA annotated T cells successfully when a smaller *k* value was used (Supplementary Figure S4). For the human PBMC dataset, scMRMA identified 3172 out of 3383 cells (93.8%), followed by SingleR for 3074 cells (90.9%). CellAssign only predicted 746 cells correctly, which labeled most cells as unassigned (Supplementary Figure S5). The human lung dataset showed high ambient RNA contamination, where highly expressed cell-type specific genes were observed in other cell populations. In this challenging case, scMRMA annotated 4596 out of 5902 cells correctly (77.9%) (Supplementary Figure S6). Compared to the manual annotation in the literature (33), we found that 391 of 804 B cells were identified as Type II alveolar cells by scMRMA. We believed that these 391 cells are truly Type II alveolar cells since they have much higher SFTPC and SFTPA2 expression than other cells. They were misclassified as B cells by the original paper because they have high expression of IGKC. However, IGKC was observed in other cells with similar abundance (Supplementary Figure S7). Therefore, the high expression of IGKC is likely due to ambient RNA contamination rather than cell-type specific expression. Among the four methods, transcriptome-based SingleR performed better than marker-based methods, Garnett, scCATCH and CellAssign. SingleR obtained high performance for cell types covered in its reference, such as endothelial, macrophage, NK and T. Its high performance likely attributes to its usage of transcriptomes as reference, which is less affected by contamination compared to the utilization of a limited number of marker genes by the other three methods. scMRMA also depends on cell-specific markers like the three methods; however, it achieved better performance than SingleR. scMRMA assigns cell annotation from broad to specific. Broad cell types include much more markers than specific ones, making them easy to be recognized. The correct assignment of broad types narrows down the prediction, leading to the robustness against contamination and improved annotation accuracy on specific cell types.

In terms of computational runtime, scMRMA, scCATCH and SingleR were comparable, whereas CellAssign required the longest runtime. Garnett was the fastest method if a pre-trained classifier was used, but its runtime was significantly increased if a classifier needs to be trained before annotations (Supplementary Table S1). All runs were conducted on a MacBook Pro Laptop equipped with 2.3 GHz Intel Core i5 CPUs at 2.3 GHz and 8 GB memory.

### The iterative cluster-and-annotate strategy improve the performance

The performance of cell type annotation depends strongly on the quality of the reference and the approach to find the best match. In addition, the iterative cluster-and-annotate from broad to specific significantly improves performance.

**Figure 4.**
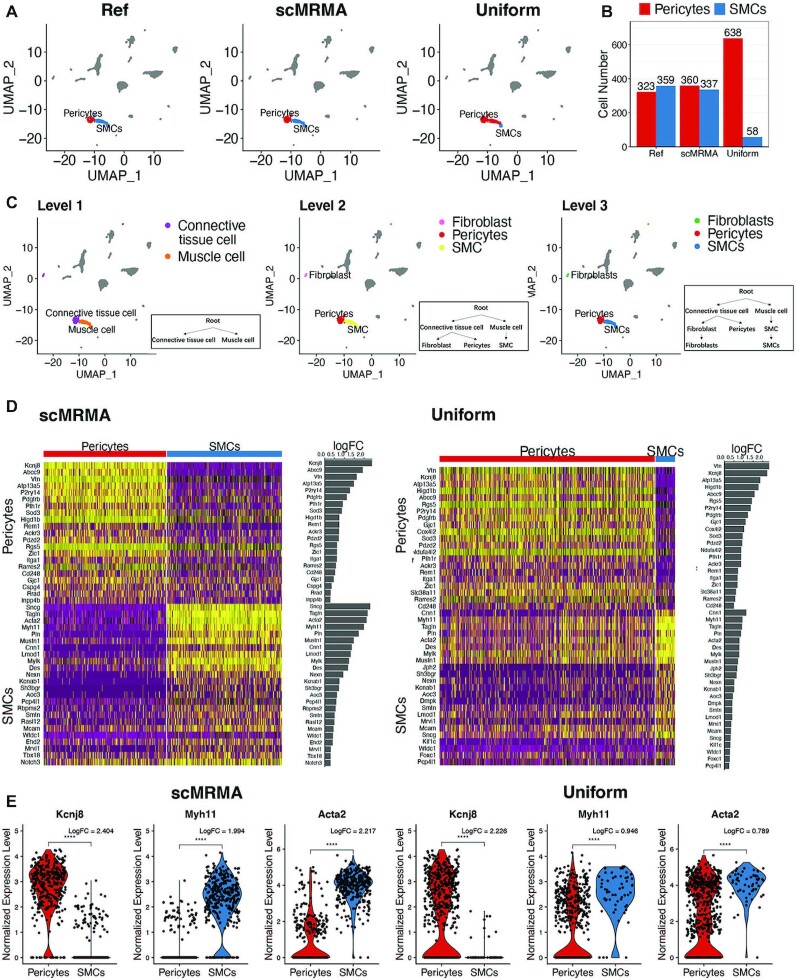
The advantage of scMRMA annotation from broad to specific. (**A**) The annotation results of the mouse brain dataset from the literature (Ref), scMRMA and Uniform. (**B**) The number of cells assigned to pericytes and SMCs by the original literature, scMRMA and Uniform. (**C**) The annotation results of scMRMA from the first level to the third level. (**D**) The heatmap and fold change of differential expressed genes between pericytes and SMCs classified by scMRMA and Uniform. (**E**) Boxplot of expression of Kcnj8, Myh11 and Acta2 between pericytes and SMCs predicted by scMRMA and Uniform.

We compared scMRMA with a method, ‘Uniform’ that groups cells at a defined resolution then assigns cell types in one step. scMRMA and Uniform used the same reference. However, scMRMA implemented iterative cluster-and-annotate, whereas Uniform did not. For the mouse brain dataset, scMRMA identified 360 pericytes and 337 Smooth Muscle Cells (SMCs), which were very close to the manual annotation in the literature (35) (Figure 4A and B). In comparison, Uniform predicted 638 Pericytes and 58 SMCs, where most SMCs were misclassified as Pericytes. The classification by scMRMA showed a more distinctive gene expression pattern between pericytes and SMCs than the one by Uniform (Figure 4D). Moreover, known Pericytes-specific or SMCs-specific markers, such as Kcnj8, Myh11 and Acta2, showed bigger difference between pericytes and SMCs in the scMRMA classification than the Uniform (Figure 4E). These observations further validated the correct annotation by scMRMA. The success of scMRMA attributes to the annotation strategy from broad to specific, where cells were correctly assigned to ‘connected tissue cell’ or ‘muscle cell’ at the first level, which avoids the misclassification between pericytes and SMC at deep levels (Figure 4C).

**Figure 5.**
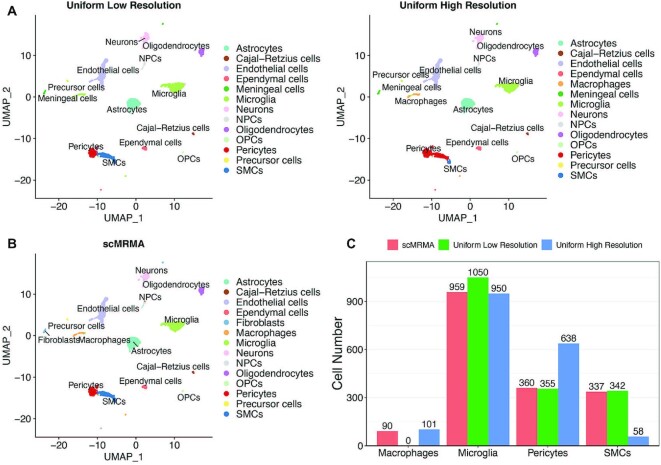
The impact of clustering resolution on scMRMA and Uniform. (**A**) The Uniform annotation results of the mouse brain dataset at a low clustering resolution and a high clustering resolution. (**B**) The annotation result of scMRMA. (**C**) The number of cells identified as macrophages, microglia, pericytes and SMCs by scMRMA, Uniform at low and high clustering resolutions.

Moreover, the performance of scMRMA is resolution-independent (Figure 5B), while a certain resolution at which Uniform grouped cells has a big impact on the annotation results. For example, Uniform identified pericytes and SMCs correctly but misclassified macrophage as microglia at a low-resolution clustering (Figure 5A and C; Supplementary Figure S8). In contrast, Uniform recognized macrophages but misclassified some pericytes at a high-resolution clustering (Figure 5A and C).

**Figure 6.**
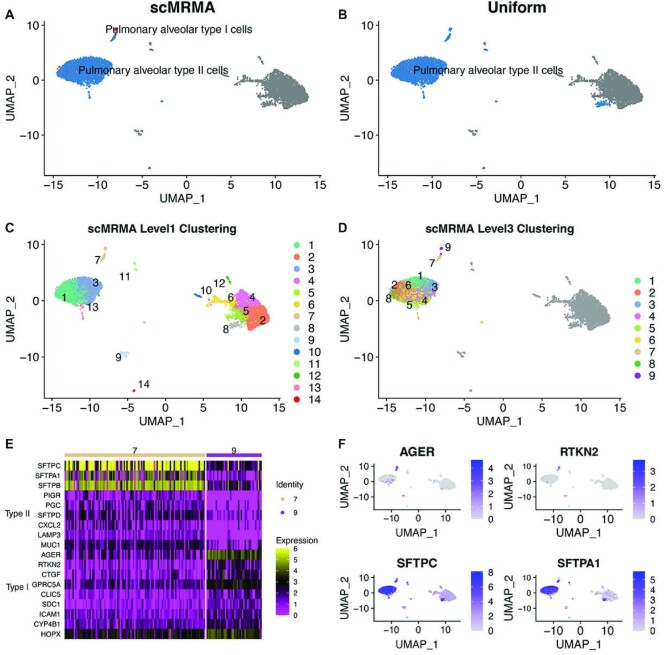
The advantage of iterative clustering of scMRMA. (**A**) The annotation results of scMRMA for the human lung dataset GSM3489182. (**B**) The Uniform annotation results. (**C**) The clustering results at the first level. (**D**) The clustering results at the third level. (**E**) The heatmap of expression of Type I (cluster 9 in Level 3) and II (cluster 7 in Level 3) alveolar specific genes. (**F**) The UMAP plot of normalized expression of AGER, RTKN2, SFTPC and SFTPA1.

In addition, the iterative clustering captures local variances in a given subpopulation, leading to improved annotation for very specific cell types. For example, pulmonary alveolar type I and type II cells were both identified by scMRMA in a human lung dataset (GSM3489182) (Figure 6A), while only pulmonary alveolar type II cells were recognized by Uniform no matter what resolutions were used (Figure 6B and Supplementary Figure S9). scMRMA identified epithelial cell (clusters 1,3, 7 and 13) and other cell types at the first level (Figure 6C). Looking into epithelial cells, scMRMA further divided the cluster 7 into two subclusters (7 and 9) (Figure 6D). The subcluster 7 was annotated as Type II alveolar cells, while the subcluster 9 was determined to be type I cells by scMRMA. The subcluster 7 showed high expression of type II markers like SFTPC, SFTPA1 and SFTPB, while the subcluster 9 had low expression of type II markers but high expression of type I markers such as AGER and RTKN2 (Figure 6E and F), which supported the annotation of scMRMA. Since the cluster of type I alveolar cells cannot be detected at any resolution by Uniform, it demonstrated the value of re-clustering, which looks into specific cell populations and captures their subtle difference. The more distinctive subcluster leads to more accurate annotation especially on very specific cell types.

### scMRMA uncovers expansion of CD8 T cells after PD-1 blockade

**Figure 7.**
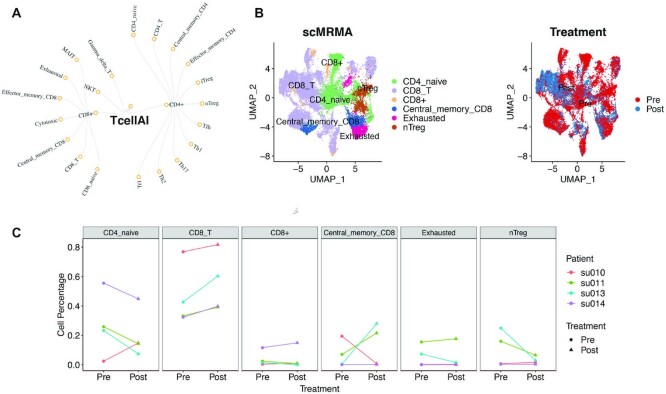
The application of scMRMA to revealing alterations in cell compositions. (**A**) The hierarchical TcellAI reference in a two-level structure. (**B**) The UMAP projection of cells colored by scMRMA annotation results. (**C**) The UMAP projection of cells colored by pre- and post-treatment. (**C**) Paired changes in cell abundance before and after treatment for each cell type. Different colors represent different patients.

With the ability of annotating cells accurately, scMRMA can estimate cell population alterations across conditions. We applied scMRMA on a scRNAseq dataset collected from four patients with squamous cell carcinoma (SCC) before and after PD-1 blockade (36). TcellAI, a collection of T cells in cancer immune environment (31), was used as the reference (details in Materials and Methods section) (Figure 7A). scMRMA found six cell types, including CD4_naive, CD8_T, CD8+, Central Memory CD8, CD8 Exhausted and nTreg (Figure 7B). Cell populations changed between pre- and post-treatment (Figure 7B). To identify the consistent changes across patients, we performed the paired analysis on the proportion of each cell type between pre-and post-treatment. We found that all four patients have expansion in CD8_T cells after PD-1 blockade (*P* = 0.1, wilcox signed-rank test) (Figure 7C). This is consistent with T-cell receptor (TCR) sequencing results, which revealed expanded clones especially in CD8 T cells (36). In comparison, the expansion of CD8 T cells was not discovered in the original manual annotation, nor detected by CellAssign, scCATCH, SingleR or Garnett (Supplementary Figure S10).

## DISCUSSION

In this study, we developed scMRMA, a multi-resolution marker-based annotation method for single cell datasets. scMRMA achieved high annotation accuracy. The success is partly attributed to the iterative cluster-and-annotate procedure, which uses a pre-defined hierarchical reference for clustering and annotates cells from broad to specific cell types.

scMRMA uses cell-specific marker genes for annotation, which is robust against technical factors or batch effects in experimental processing. Cell assignment from broad to specific cell types make it highly tolerant to technical factors like ambient RNA contamination since broad cell types are very distinct and easily recognized. The correct assignment to broad cell types narrows down the prediction and improves annotation accuracy. Moreover, scMRMA captures both global and local variances of diverse cell populations through the iterative cluster-and-annotate procedure. The number of clustering steps depends on the depth of the hierarchical reference. When one cell type has more than one specific subtypes, scMRMA will take a closer look into cells belonging to the cell type, trying to capture the subtle difference and to map them to specific subtypes accurately. This procedure improves clustering performance on specific cell types, leading to distinctive subclusters and accurate annotation.

The performance of automatic cell annotation methods highly depends on the quality of the reference. They would likely to fail if cells are unknown to their reference (poorly characterized cell types). Although scMRMA requires prior knowledges of cell-specific marker genes, it can assign those unknown cells to broad types even if it cannot annotate them very specifically. As the knowledge on cell types increases over time, scMRMA can easily add them into the hierarchical reference by expanding its breadth and depth. scMRMA intrinsically handles the challenge caused by highly specific cell types.

## DATA AVAILABILITY

The scMRMA R package is freely available in the Github repository (https://github.com/JiaLiVUMC/scMRMA).

Accession numbers for four benchmark datasets the mouse brain, the human pancreas, PBMC and lung datasets include GSM3580745, GSE84133, GSM2486333 and GSE130148. Accession number for one dataset used for T cell type change application is GSE123813.

## Supplementary Material

gkab931_Supplemental_File
