# Supplementary material for: scMRMA: single cell multiresolution marker-based annotation
Source: Nucleic Acids Res. 2021 Oct 14;50(2):e7. doi: 10.1093/nar/gkab931 (PMC8789072; doi:10.1093/nar/gkab931)
Supplement: gkab931_Supplemental_File [file gkab931_supplemental_file.pdf]

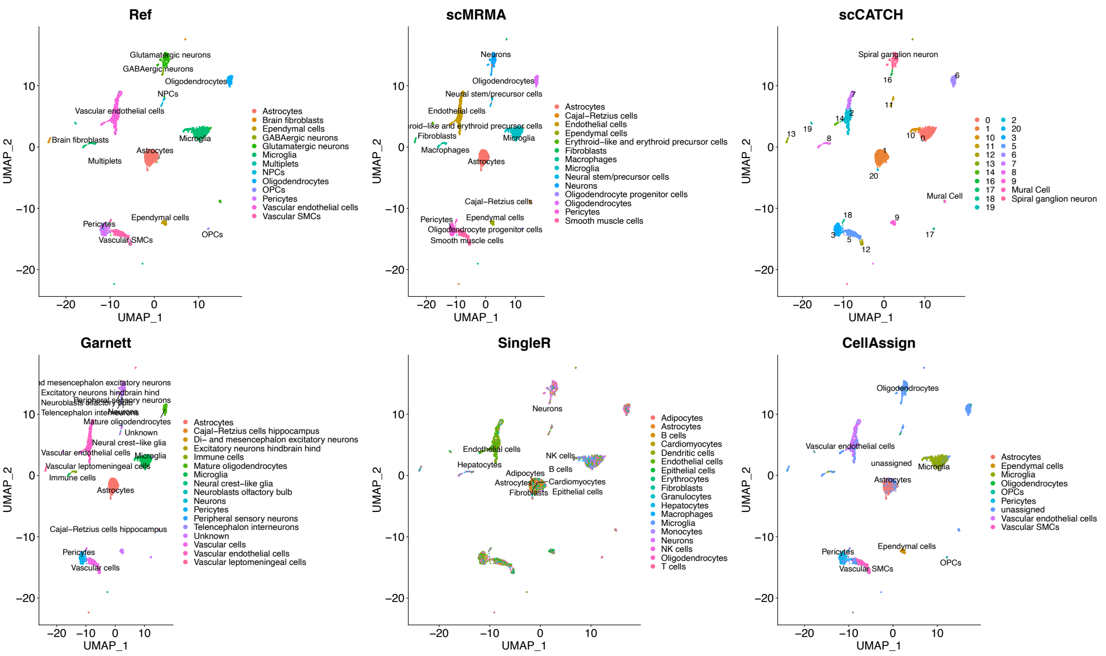

Figure S1 Annotation results of five methods for the mouse brain dataset. UMAP projection of mouse brain dataset (n= 3,985) annotated by the literature, scMRMA, scCATCH, Garnett, SingleR, and CellAssign.

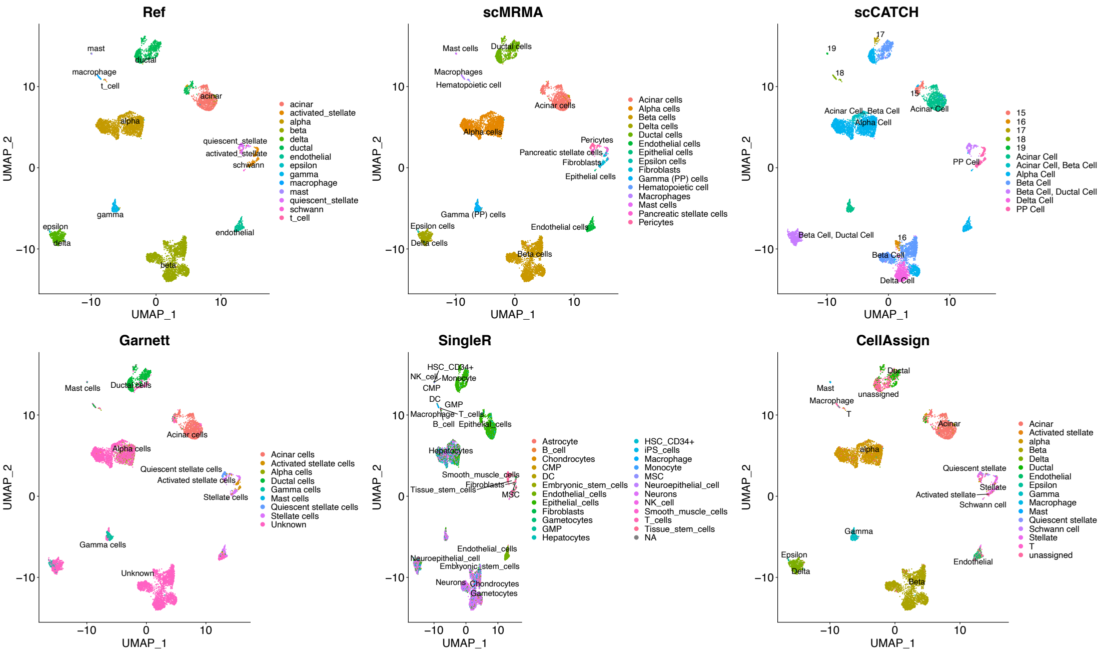

Figure S2 Annotation results of five methods for the human pancreas dataset. UMAP projection of human pancreas dataset (n= 8,569) annotated by the literature, scMRMA, scCATCH, Garnett, SingleR, and CellAssign.

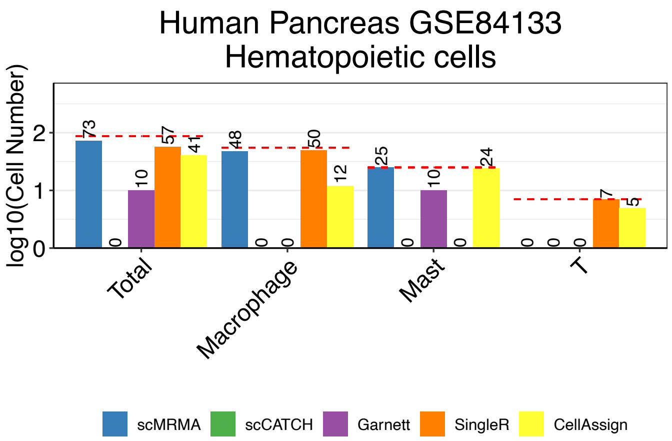

Figure S3 Comparison of annotation accuracy on Hematopoietic cells from human pancreas dataset. The "Gold-standard" annotation from the literature was represented by red dashed lines.

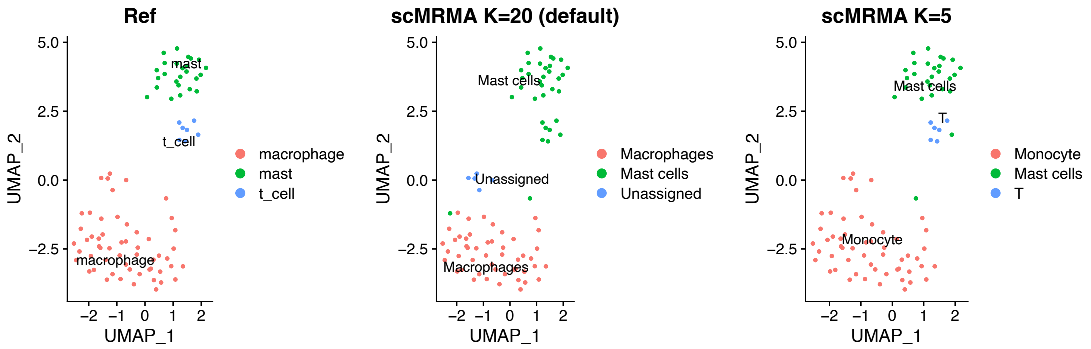

Figure S4 Hematopoietic cells from human pancreas dataset annotated with different K value. K is the parameter to find the k-nearest neighbor, default is 20.

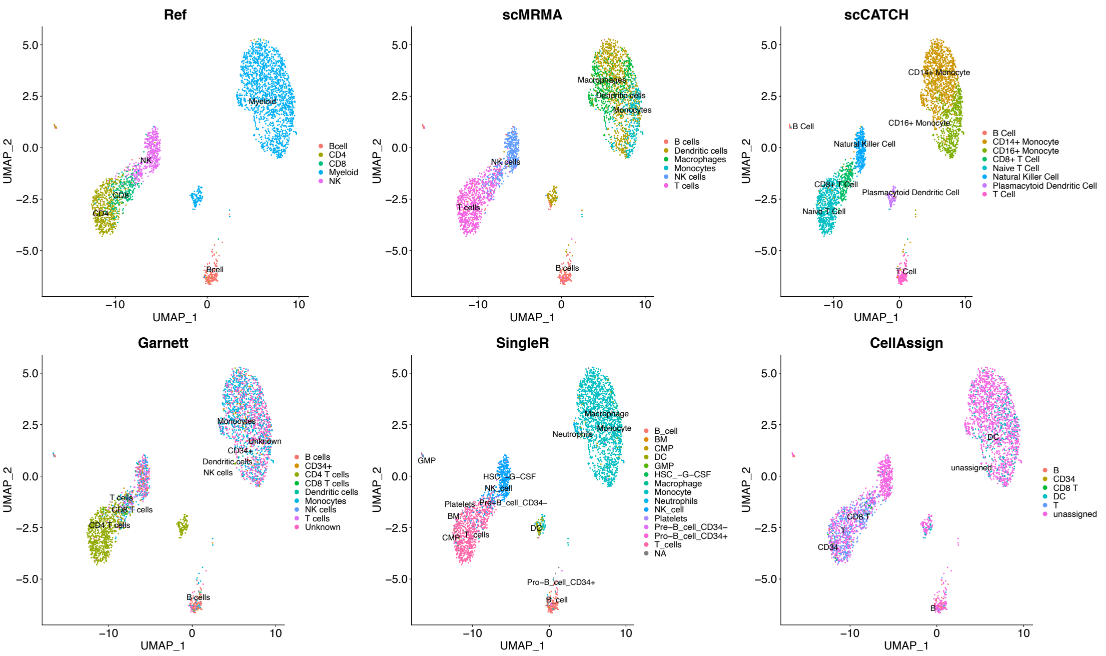

Figure S5 Annotation results of five methods for the human PBMC dataset. UMAP projection of human PBMC dataset (n= 3,383) annotated by the literature, scMRMA, scCATCH, Garnett, SingleR, and CellAssign.

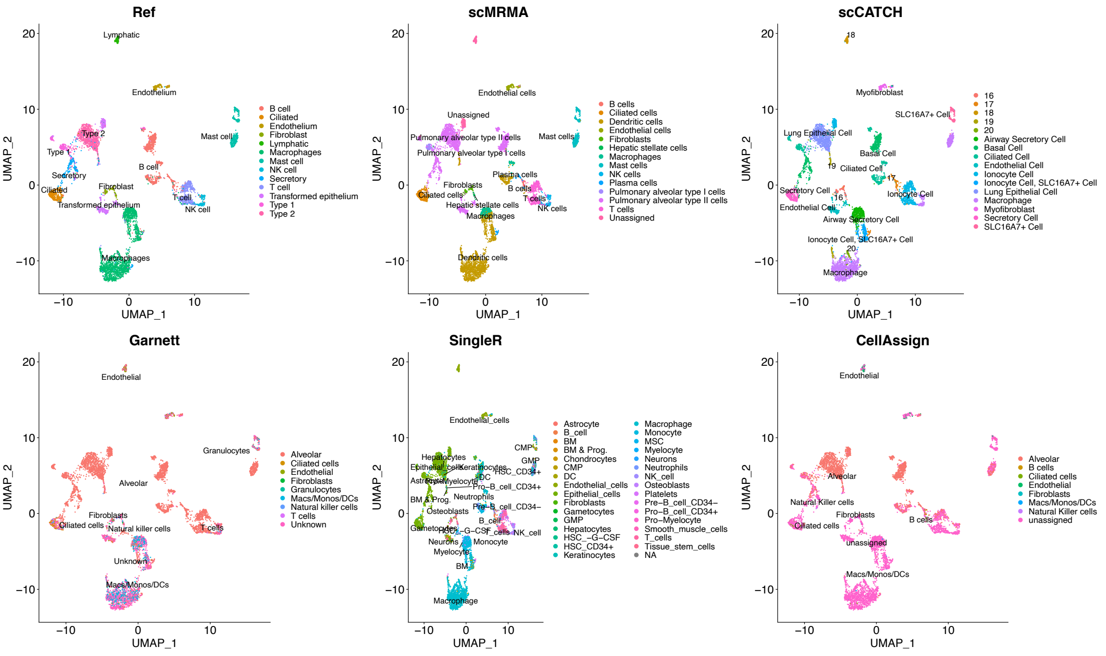

Figure S6 Annotation results of five methods for the human lung dataset. UMAP projection of human lung dataset (n= 6,007) annotated by the literature, scMRMA, scCATCH, Garnett, SingleR, and CellAssign.

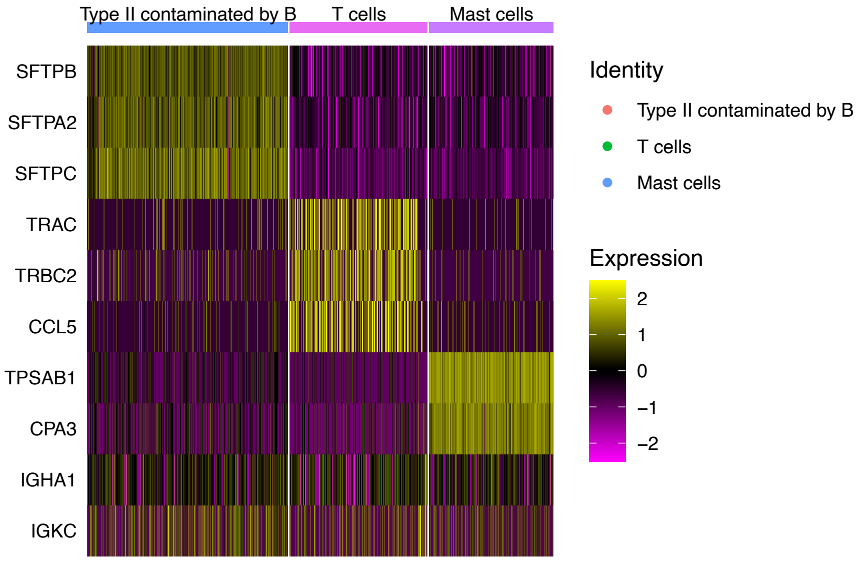

Figure S7 Heatmap of markers in Type II alveolar cells, T cells and mast cells. IGHA1 and IGKC are markers from B cells.

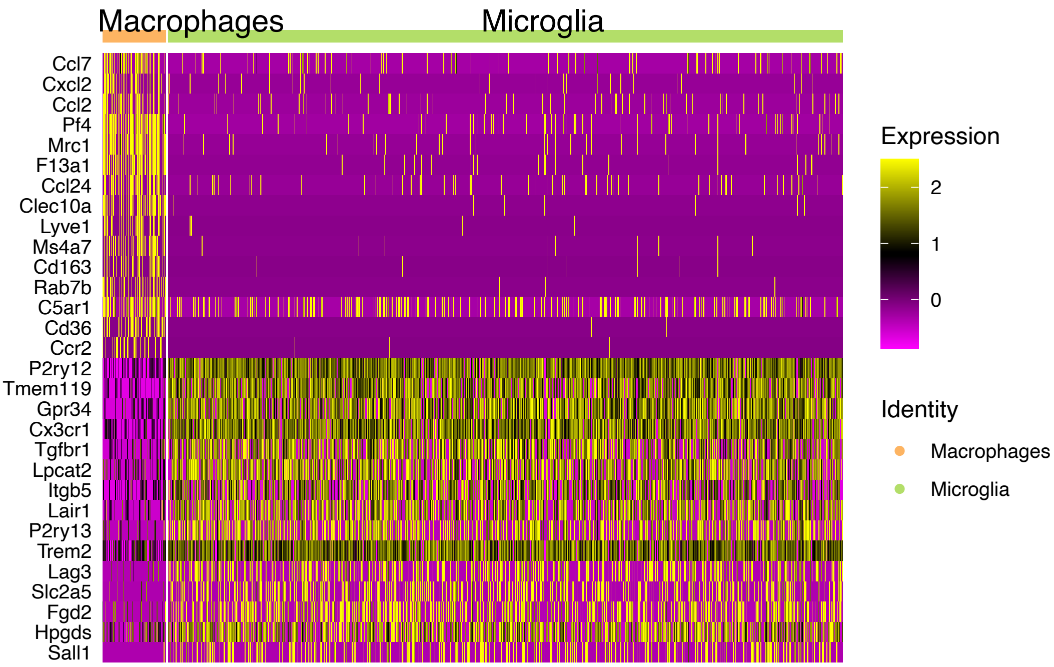

Figure S8 Heatmap of differential expressed genes between Macrophages and Microglia in the mouse brain dataset.

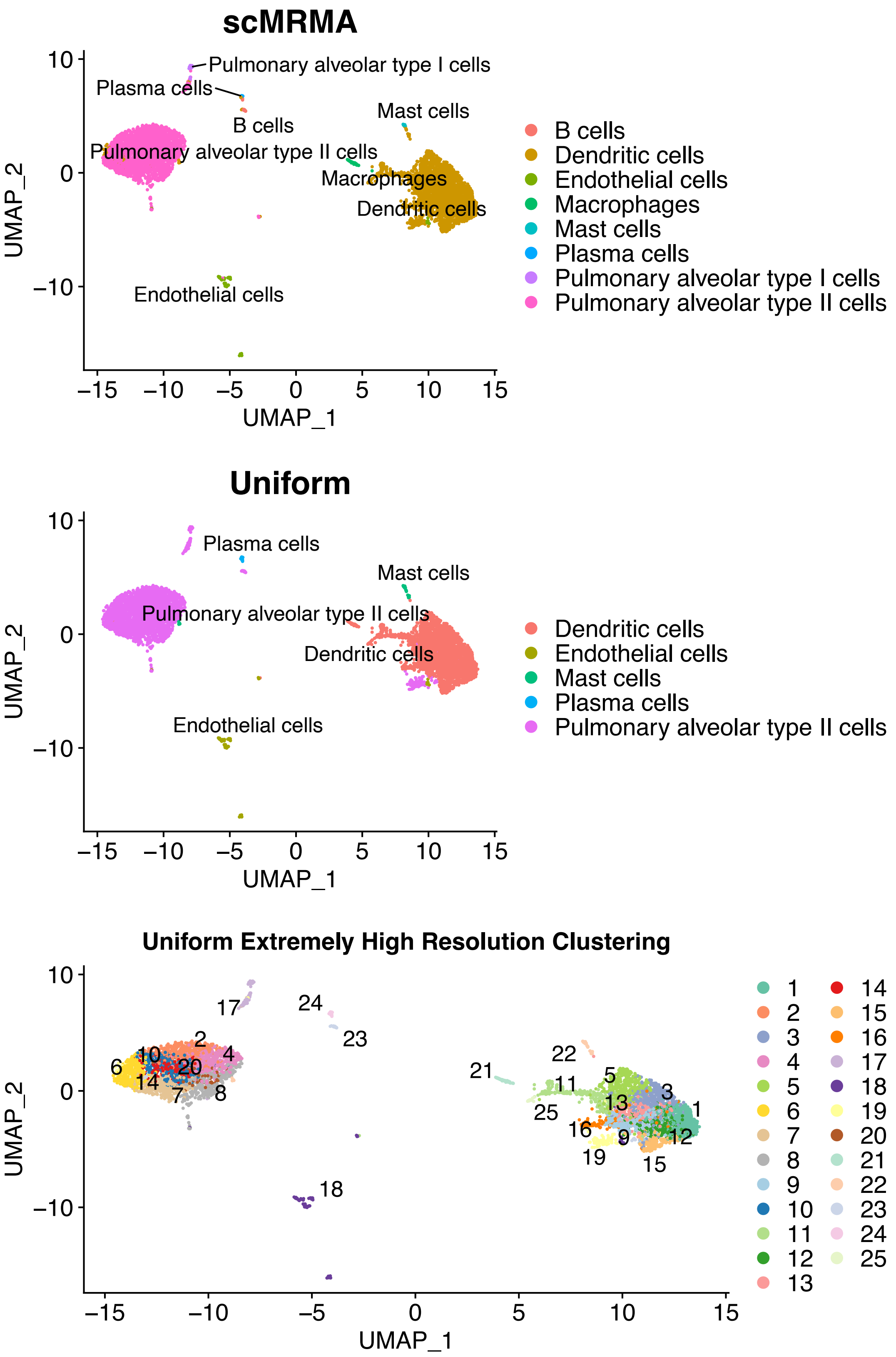

Figure S9 Annotation results of scMRMA and Uniform in human lung dataset GSM3489182. Clustering result with extremely high clustering resolution.

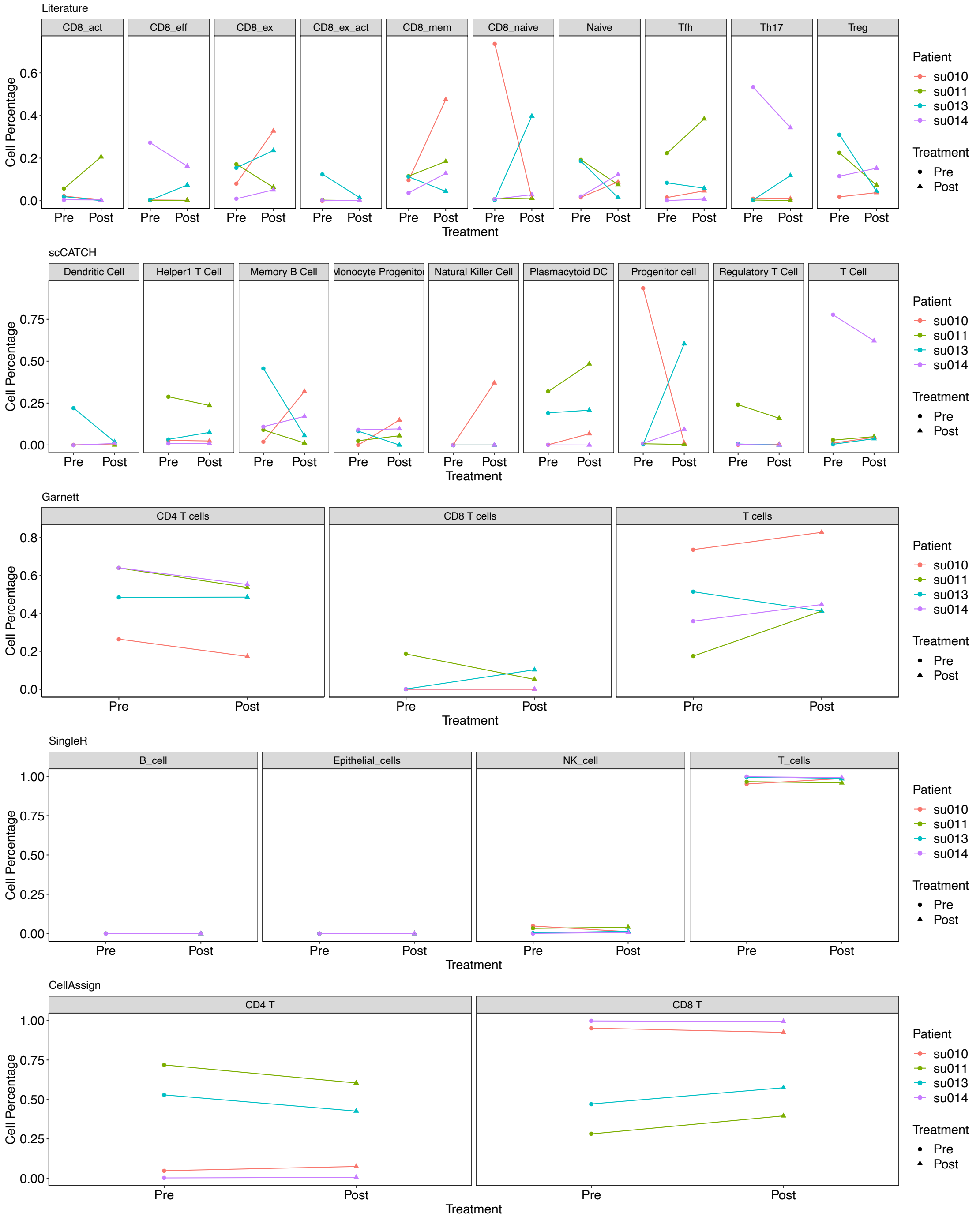

Figure S10 Cell population changes between pre- and post-treatment by the literature, Garnett, CellAssign, scCATCH and SingleR. Different colors represent different patients.

Supplementary Table 1 The computational runtime

Dataset	scMRMA	scCATCH	Garnett	SingleR	CellAssign
**Mouse brain GSM3580745**	118s	12s	47s*	7s	>30mins
**Human pancreas GSE84133**	108s	152s	>30mins	459s	>30mins
**Human PBMC GSM2486333**	115s	118s	8s*	44s	630s
**Human lung GSE130148**	246s	536s	11s*	253s	>30mins
**Human SCC GSE123813**	424s	1376s	88s*	163s	>30mins

** use pre-trained classifier. The runtime of SingleR highly depends on which reference datasets are used.*
